# The impact of pre-existing hypertension and its treatment on outcomes in patients admitted to hospital with COVID-19

**DOI:** 10.1038/s41440-022-00893-5

**Published:** 2022-03-29

**Authors:** Ewan McFarlane, Marijke Linschoten, Folkert W. Asselbergs, Peter S. Lacy, Dawid Jedrzejewski, Bryan Williams, on Behalf of the CAPACITY-COVID Consortium

**Affiliations:** 1 Institute of Cardiovascular Science, Faculty of Population Health Sciences, University College London, London, UK; 2 Department of Cardiology, Division of Heart and Lungs, University Medical Center Utrecht, Utrecht University, Utrecht, the Netherlands; 3 National Institute for Health Research Biomedical Research Center, University College London Hospitals, London, UK; 4 Health Data Research United Kingdom and Institute of Health Informatics, University College London, London, UK

**Keywords:** Hypertension, SARS-Cov-2, COVID-19, Public health

## Abstract

The impact of pre-existing hypertension on outcomes in patients with the novel corona virus (SARS-CoV-2) remains controversial. To address this, we examined the impact of pre-existing hypertension and its treatment on in-hospital mortality in patients admitted to hospital with Covid-19. Using the CAPACITY-COVID patient registry we examined the impact of pre-existing hypertension and guideline-recommended treatments for hypertension on in-hospital mortality in unadjusted and multi-variate-adjusted analyses using logistic regression. Data from 9197 hospitalised patients with Covid-19 (median age 69 [IQR 57–78] years, 60.6% male, *n* = 5573) was analysed. Of these, 48.3% (*n* = 4443) had documented pre-existing hypertension. Patients with pre-existing hypertension were older (73 vs. 62 years, *p* < 0.001) and had twice the occurrence of any cardiac disease (49.3 vs. 21.8%; *p* < 0.001) when compared to patients without hypertension. The most documented class of anti-hypertensive drugs were angiotensin receptor blockers (ARB) or angiotensin converting enzyme inhibitors (ACEi) (*n* = 2499, 27.2%). In-hospital mortality occurred in (*n* = 2020, 22.0%), with more deaths occurring in those with pre-existing hypertension (26.0 vs. 18.2%, *p* < 0.001). Pre-existing hypertension was associated with in-hospital mortality in unadjusted analyses (OR 1.57, 95% CI 1.42,1.74), no significant association was found following multivariable adjustment for age and other hypertension-related covariates (OR 0.97, 95% CI 0.87,1.10). Use of ACEi or ARB tended to have a protective effect for in-hospital mortality in fully adjusted models (OR 0.88, 95% CI 0.78,0.99). After appropriate adjustment for confounding, pre-existing hypertension, or treatment for hypertension, does not independently confer an increased risk of in-hospital mortality patients hospitalized with Covid-19.

## Graphical abstract

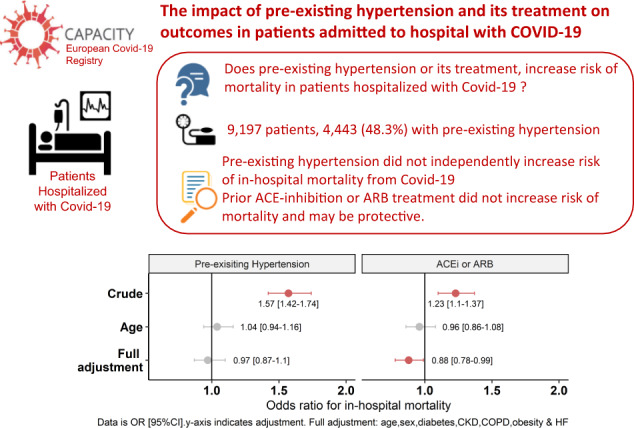

## Introduction

Soon after the emergence of the novel coronavirus (SARS-CoV-2), first reported in December 2019, it was widely reported that hypertension was a major risk factor for developing more severe Covid-19, i.e. the need for hospitalization, intensive care, and/or risk of death from this disease [1, 2]. This led to speculation that this hypertension-associated increased risk might, at least in part, be due to the most common drugs used to treat hypertension, notably; renin-angiotensin system (RAS) inhibitors, such as angiotensin converting enzyme inhibitors (ACEi) or angiotensin receptor blockers (ARB) [3–5]. This therapeutic link was hypothesized based on the observation that SARS-CoV-2 enters human cells via ACE-2, citing previous studies suggesting that treatment with RAS-inhibitors might lead to the increased cellular expression of ACE-2 [6]. This led to global anxiety and concern amongst doctors and their patients.

Since then, pre-existing hypertension has been consistently reported to be the most common comorbidity in patients hospitalized with Covid-19 [7–12]. The challenge with early reports associating hypertension with the risk of severe Covid-19 or death was that all other risk factors for adverse outcomes from Covid-19 were not fully elucidated at the time. We now know that men, the elderly, and those with multiple comorbidities, especially diabetes, are at higher risk of severe disease and mortality from Covid-19 [7, 13–15]. Thus, finding that hypertension is associated with poorer outcomes from Covid-19 is not surprising because hypertension is very common, with a global prevalence of ~30% [16] in the general population, and a prevalence that increases markedly with age. Moreover, hypertension often complicates or co-segregates with other comorbidities such as obesity, diabetes, chronic kidney disease and cardiovascular diseases (especially heart failure), all of which are known to increase risk in hospitalised patients with Covid-19.

The association of pre-existing hypertension with risk of mortality due to Covid-19, consistently shows a two-to-four-fold greater odds ratio (OR) for a worse outcome [1, 10–12, 14, 17, 18] in unadjusted analyses. In some studies, hypertension remains a significant risk factor for mortality due to Covid-19 even after adjustment for other comorbidities [1, 17]. In contrast, a large study from Italy (n ~4000), reported that pre-existing hypertension was not associated with worse survival probability in patients requiring intensive care [18]. Furthermore, a very large study from the United Kingdom (UK), using over 17 million patients’ data from general practices, showed that high blood pressure, or a diagnosis of hypertension, was associated with a slight increase Covid-19 related in-hospital mortality following age and sex adjustment, but not after full adjustment [15]. Thus, it remains controversial and unclear [19] whether there is an independent effect of pre-existing hypertension on Covid-19 outcomes [5].

Reflecting the concern, the World Health Organisation (WHO) recently issued a scientific brief that concluded that almost all available evidence suggests that hypertension increases the risk of severe Covid-19 [20]. This WHO scientific brief acknowledged, however, that many of the early reports were unadjusted for potential confounding and further studies were needed to better clarify the relationship between pre-existing hypertension and outcomes from Covid-19. To address this, our primary aim was to examine the effect of pre-existing hypertension on in-hospital mortality with appropriate adjustment for confounders, using a large European, academically-led, patient registry: CAPACITY-COVID [21, 22] designed to evaluate the impact of cardiovascular risk factors and disease on clinical outcomes in patients hospitalised with Covid-19. In secondary analysis, we also examined the association between pre-existing treatment with different anti-hypertensive medications and in-hospital mortality.

## Methods

### Data set

We used data from the CAPACITY-COVID patient registry (www.capacity-covid.eu; NCT04325412) that was purposely designed to collate detailed information regarding cardiovascular risk factors and complications from Covid-19 during hospital admission. The details of this registry have been previously outlined [21, 22]. In short, all adult patients (≥18 years) with highly suspected or laboratory confirmed SARS-CoV-2 infection that required hospitalization were eligible for inclusion in the registry. The Case Record Form (CRF) released by the International Severe Acute Respiratory and Emerging Infection Consortium (ISARIC) and WHO in response to the emerging outbreak of Covid-19 was used as the foundation but extended with ~400 additional data fields to capture detailed information regarding cardiovascular risk factors, the use of cardiovascular medication and cardiovascular outcomes. The informed consent procedure varied per study site, following local and national rules and regulations during the pandemic, and complied with the Declaration of Helsinki. Some participating sites used an opt-out approach, where patients received written information during or after hospital admission. For sites in the UK, informed consent was not required under emergency legislation during the pandemic. Participating centres uploaded data in a pseudonymised format to a cloud-based REDCap data repository, which was managed by the University Medical Centre Utrecht, Utrecht, the Netherlands. Raw data was made available for analysis in a secure cloud-based interface.

### Participant selection criteria

Participants were included in the present analysis based on the following criteria: (1) SARS-CoV-2 infection confirmed by either a positive swab at/or during hospital admission, or highly suspected based on clinical criteria early in the pandemic before routine testing had been established; (2) documentation of pre-existing hypertension status in the data record; (3) ≥18 years, where age was available; (4) known outcome at discharge from hospital and (5) documented date of hospital admission between 1st March 2020 and 18th April 2021.

### Statistical analyses

The primary analysis assessed the association between pre-existing hypertension and in-hospital mortality using logistic regression models. “No in-hospital mortality” was defined as a composite of discharged alive, transfer to another facility or palliative discharge. In exploratory analysis of other pre-existing comorbidities, variables that were independently associated [i.e OR and 95% confidence interval (CI) not spanning (1)] with in-hospital mortality after adjustment for age and sex, were retained for fully adjusted models. The variables used in fully adjusted models included; age, sex, diabetes, chronic kidney disease (documented in the EHR, or using eGFR <60 mL/min/1.73 m^2^ or urine albumin-creatinine ratio ≥3 mg/mmol), chronic obstructive pulmonary disease (documented in the EHR, or using cut-off values defined as: FEV1 / FVC < 5th percentile of reference population (z-score < −1.64)), obesity (body mass index ≥30 kg/m^2^) and known heart failure. These variables were used in both the primary and secondary analyses.

In secondary analysis, the effects on outcomes of 5 types of guideline-recommended and commonly used anti-hypertensive medications (ACEi, ARB, pooled ACEi or ARB, beta–blockers (BB), calcium channel blockers (CCB) and diuretics (D)) were examined using logistic regression models, unadjusted and with the following three sequential statistical adjustments (age adjusted, age and sex adjusted, and fully adjusted). Two sensitivity analyses were also performed following; (1) the exclusion of individuals who did not have a laboratory confirmed SARS-CoV-2 infection at or during hospital admission (*n* = 946, 10.3%) but were highly suspected to have COVID-19 based on clinical criteria, and (2) the exclusion of individuals without documented pre-existing hypertension but who were taking medications commonly used to treat hypertension (e.g. ACEi or ARBs, BB and/or D) (*n* = 985, 10.7%), but most likely for reasons other than hypertension, e.g., heart failure, diabetes and chronic kidney disease.

For participants with both a SARS-CoV-2 infection confirmed by either a positive swab at/or during the hospital admission, or highly suspected based on clinical criteria and documentation of pre-existing hypertension status in the data record (*n* = 9197), the missing data for age (*n* = 89) was imputed using the median age, based on pre-existing hypertension status. Similarly, missing values for sex (*n* = 122) were imputed using the most common class by pre-existing hypertension status. Variables with missing data that were retained from exploratory analysis for use in fully adjusted models were imputed using a random forest algorithm, the imputation errors are shown in (Supplementary Table 1) where the maximum number of iterations was 5 and number of trees 20.

Data tables were stratified by pre-existing hypertension with continuous variables compared using the Wilcoxon rank sum test and categorical variables using the Chi-square test. Data in tables are displayed as N (%) for categorical variables and presented as the proportion of the column variable total, or median (interquartile range) for continuous variables. Logistic regression models are reported as OR or adjusted OR (aOR) [95% CI]. R studio version 4.0.4 was used for all analyses and the gtsummary, ggplot2 and missForest packages were used to create data tables, figures and imputation, respectively.

## Results

**Fig. 1:**
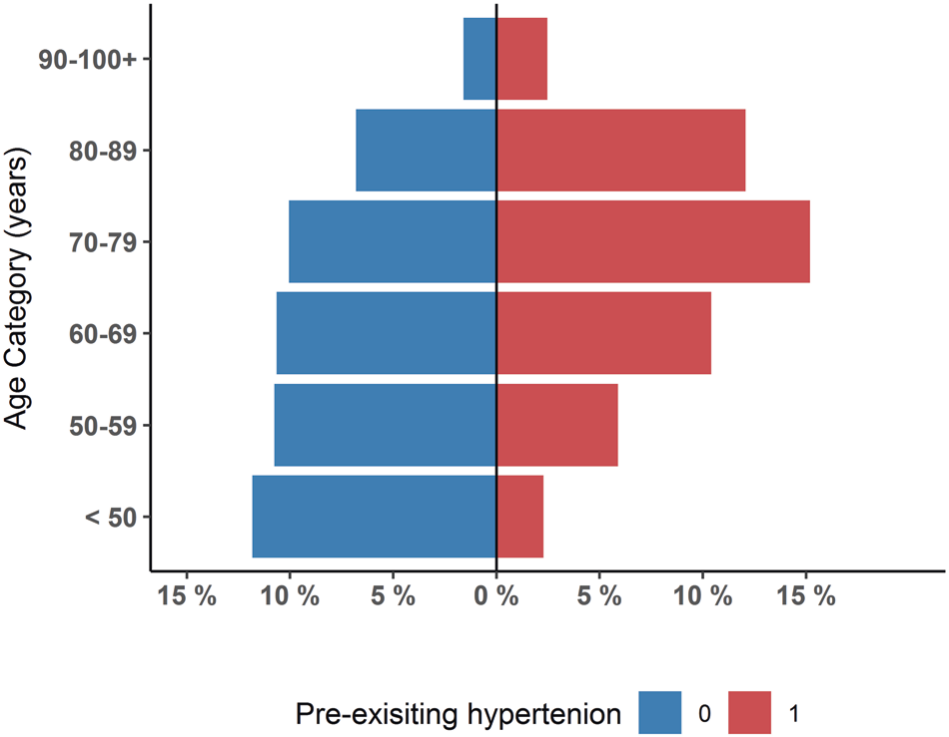
Distribution of pre-existing hypertension by age group as a proportion of total sample *N* = 9197. 0 blue: indicates no documented pre-existing hypertension; 1 red: indicates documented pre-existing hypertension

The data set comprised of 12,227 individuals with outcome data of whom 9197 were included in the study based on pre-defined criteria. Demographic characteristics are shown in (Table 1). In the overall population (*n* = 9197), there was a greater proportion of males (*n* = 5573, 60.6%), median age was 69 years, and the majority were white (*n* = 6313; 68.6%). In total, (*n* = 4443, 48.3%) of patients had pre-existing hypertension and the hypertensive cohort was older, had a higher body mass index and a greater burden of obesity, cardiovascular disease, chronic lung disease, diabetes and dyslipidemia, compared to those without pre-existing hypertension. Imputation errors and missingness of data is reported in (Supplementary Table 1) and study demographics stratified by pre-existing hypertension with the imputed data is shown in (Supplementary Table 2). When stratified by pre-existing hypertension, age groups below the group 60–69 years had fewer patients with pre-existing hypertension, conversely the opposite trend was seen in those above this age group (Fig. 1).

**Table.1:** Demographics, stratified by pre-existing hypertension

		Pre-existing hypertension		
Characteristic	Overall, *N* = 9197	No, *N* = 4758 (51.7%)	Yes, *N* = 4443 (48.3%)	*p* value
Sex, Male	5573 (60.6%)	2996 (63.0%)	2577 (58.0%)	<0.001
Age, Years	69 (57, 78)	62 (51, 74)	73 (63, 81)	<0.001
Age Group, Years				<0.001
<50	1298 (14.1%)	1088 (22.9%)	210 (4.7%)	
50–59	1531 (16.6%)	989 (20.8%)	542 (12.2%)	
60–69	1937 (21.1%)	980 (20.6%)	957 (21.5%)	
70–79	2321 (25.2%)	924 (19.4%)	1397 (31.4%)	
80–89	1736 (18.9%)	626 (13.2%)	1110 (25.0%)	
90–100+	374 (4.1%)	147 (3.1%)	227 (5.1%)	
Body mass index (BMI), kg/m^2^	27.2 (24.2, 30.9)	26.8 (23.9, 30.4)	27.8 (24.6, 31.6)	<0.001
Obese, BMI ≥ 30 kg/m^2^	1877 (30.7%)	832 (26.9%)	1045 (34.7%)	<0.001
Heart disease- any type	3208 (35.1%)	1028 (21.8%)	2180 (49.3%)	<0.001
Arrhythmia or conduction disorder	1392 (15.2%)	492 (10.4%)	900 (20.3%)	<0.001
Heart failure	667 (7.3%)	179 (3.8%)	488 (11.0%)	<0.001
NYHA class				0.4
NYHA I/IV	29 (4.3%)	7 (3.9%)	22 (4.5%)	
NYHA II/IV	124 (18.6%)	25 (14.0%)	99 (20.3%)	
NYHA III/IV	70 (10.5%)	20 (11.2%)	50 (10.2%)	
NYHA IV/IV	19 (2.8%)	5 (2.8%)	14 (2.9%)	
Unknown NYHA	425 (63.7%)	122 (68.2%)	303 (62.1%)	
Coronary artery disease	1356 (14.8%)	408 (8.6%)	948 (21.5%)	<0.001
Valvular heart disease	408 (4.5%)	143 (3.0%)	265 (6.0%)	<0.001
Congenital heart disease	35 (0.4%)	17 (0.4%)	18 (0.4%)	0.7
Other heart disease	466 (5.1%)	114 (2.4%)	352 (8.0%)	<0.001
Chronic Kidney Disease	1123 (12.3%)	290 (6.2%)	833 (18.9%)	<0.001
Chronic Kidney Disease - Severity				>0.9
Mild	197 (17.5%)	52 (17.9%)	145 (17.4%)	
Moderate	353 (31.4%)	91 (31.4%)	262 (31.5%)	
Severe	381 (33.9%)	95 (32.8%)	286 (34.3%)	
Unknown severity	192 (17.1%)	52 (17.9%)	140 (16.8%)	
Peripheral artery disease	359 (5.1%)	115 (3.1%)	244 (7.4%)	<0.001
Chronic obstructive pulmonary disease	1052 (11.6%)	450 (9.6%)	602 (13.7%)	<0.001
Diabetes	2391 (26.2%)	764 (16.1%)	1627 (37.0%)	<0.001
Dyslipidemia	2973 (34.3%)	845 (18.7%)	2128 (51.5%)	<0.001
Ethnicity				<0.001
Arab	494 (5.4%)	290 (6.1%)	204 (4.6%)	
Asian	623 (6.8%)	358 (7.5%)	265 (6.0%)	
Black	292 (3.2%)	141 (3.0%)	151 (3.4%)	
Latin American	23 (0.3%)	11 (0.2%)	12 (0.3%)	
Other	488 (5.3%)	304 (6.4%)	184 (4.1%)	
Unknown	964 (10.5%)	541 (11.4%)	423 (9.5%)	
White	6,313 (68.6%)	3109 (65.4%)	3204 (72.1%)	

*p* value is for comparison between pre-existing hypertension and no pre-existing hypertension documented, using Wilcoxon rank sum test or Chi-squared test

Chronic kidney disease severity: mild (eGRF 45–49,ACR 3–29), moderate (eGFR:30–44 and ACR < 3, or eGFR 45–59 and ACR 3–30, or eGFR ≥ 60 and ACR > 30), severe (eGFR < 30, or eGFR 30–44 and ACR 3–30, or eGFR 45–59 and ACR > 30)

*ACR* albumin-creatinine ratio (mg/mmol), *BMI* body mass index, *NYHA* New York heart association for classification of heart failure severity, *eGFR* estimated glomerular filtration ration (mL/min/1.73 m^2^)

The most common symptoms documented at admission to hospital by all participants were a fever (*n* = 5611, 61.0%), dyspnoea (*n* = 5276, 57.4%), cough (*n* = 5206, 56.6%), and fatigue (*n* = 2922, 31.8%) (Supplementary Table 3). There was a tendency for these symptoms to occur more frequently in those without pre-existing hypertension, i.e., younger patients. For all other symptoms documented, these occurred in <25% of the whole sample. In less frequently reported symptoms (<10% of all), a sore throat (8.2 vs. 6.5%; *p* = 0.002) was less common and peripheral oedema (0.4 vs. 1.3%; *p* < 0.001) more common in pre-existing hypertensives.

Blood pressure (BP) measured at hospital admission was higher in pre-existing hypertensive patients for systolic BP (131 vs. 129 mmHg; *p* < 0.001) but not diastolic BP (Table 2). Blood tests taken within 24 h of admission to hospital showed similar values for C-reactive protein and platelets (all; *p* > 0.05) whereas white blood cell count was marginally higher and haemoglobin marginally lower in those with pre-existing hypertension.

**Table 2:** Vital signs at admission to hospital and blood tests taken within 24 h of admission

			Pre-existing hypertension		
Characteristic	*N*	Overall, *N* = 9197	No, *N* = 4754 (51.7%)	Yes, *N* = 4443 (48.3%)	*p* value
Systolic blood pressure, mmHg	7384	131 (117, 146)	129 (116, 143)	134 (119, 150)	<0.001
Diastolic blood pressure, mmHg	7384	76 (66, 85)	76 (67, 85)	76 (66, 86)	0.6
Pulse, beats/min	7469	89 (77, 101)	90 (79, 102)	88 (76, 100)	<0.001
Respiratory rate, breaths/min	7192	21 (18, 25)	20 (18, 25)	21 (18, 25)	0.8
Oxygen saturation, %	7466	95 (93, 97)	95 (93, 97)	95 (92, 97)	<0.001
Temperature, °C	7387	37.50 (36.80, 38.30)	37.60 (36.80, 38.40)	37.50 (36.70, 38.30)	<0.001
C-reactive protein, mg/dL	7531	82 (36, 154)	83 (37, 156)	82 (36, 151)	0.2
Haemoglobin, mmol/L	7597	8.20 (7.30, 9.00)	8.30 (7.40, 9.10)	8.10 (7.20, 8.90)	<0.001
Platelets, 10^9/L	7215	211 (162, 278)	213 (163, 276)	209 (160, 279)	0.5
White blood cells, 10^9/L	7694	7.1 (5.1, 9.8)	7.0 (5.0, 9.6)	7.2 (5.3, 10.1)	<0.001

*p* value is for comparison between pre-existing hypertension and no pre-existing hypertension documented, using Wilcoxon rank sum test

### Anti-hypertensive Medications

There were five types of anti-hypertensive medications documented (ACEi, ARB, BB, CCB and D), details of specific names of these medications are available in the data supplement (Supplementary Table 4). Participants with pre-existing hypertension were most likely to be taking 1 or 2 BP-lowering medications, with RAS inhibitors (*n* = 2096) being taken most frequently, followed by BB (*n* = 1820), D (*n* = 1443) and CCB (*n* = 1324) (Table 3). For some individuals with pre-existing hypertension, they were not receiving anti-hypertensive medication, or it was not documented in this sub-group (*n* = 858, 19.3%). Conversely, a similar proportion of those without documented pre-existing hypertension (*n* = 985, 10.7%) were receiving a class of anti-hypertensive medication. In this sub-group, the majority were receiving a BB (*n* = 571; 12.0%), ACEi or ARB (*n* = 403; 8.5%) or D (*n* = 363; 7.6%) with a very small number receiving a CCB (*n* = 174; 3.7%). It is likely that these medications were being prescribed for cardiovascular disease, diabetes, or renal diseases independent of hypertension. Consistent with this, these individuals were older (74 vs. 59 years; *p* < 0.001) and had a greater burden of pre-existing cardiac disease (67.1 vs. 9.7%; *p* < 0.001) and multiple comorbidities compared to those without pre-existing hypertension who were documented as not receiving any class of anti-hypertensive medication (Supplementary Table 5). In those without pre-existing hypertension, older individuals (>69 years) accounted for most cases that were receiving a class of anti-hypertension medication (Supplementary Table 6).

**Table 3:** Anti-hypertensive medications, stratified by pre-existing hypertension

		Pre-existing hypertension		
Characteristic	Overall, *N* = 9197	No, *N* = 4754 (51.7%)	Yes, *N* = 4443 (48.3%)	*p* value
Prescribed anti-hypertensive, Yes	4570 (49.7%)	985 (20.7%)	3,585 (80.7%)	<0.001
Number of anti-hypertensives				<0.001
0	4627 (50.3%)	3769 (79.3%)	858 (19.3%)	
1	1988 (21.6%)	569 (12.0%)	1419 (31.9%)	
2	1666 (18.1%)	313 (6.6%)	1353 (30.5%)	
3 or more	916 (10.0%)	103 (2.2%)	813 (18.3%)	
ACEi or ARB	2499 (27.2%)	403 (8.5%)	2096 (47.2%)	<0.001
Beta-blocker	2391 (26.0%)	571 (12.0%)	1820 (41.0%)	<0.001
Diuretic	1806 (19.6%)	363 (7.6%)	1443 (32.5%)	<0.001
CCB	1498 (16.3%)	174 (3.7%)	1324 (29.8%)	<0.001
ACEi	1512 (16.4%)	286 (6.0%)	1226 (27.6%)	<0.001
ARB	1007 (10.9%)	118 (2.5%)	889 (20.0%)	<0.001

*p* value is for comparison between pre-existing hypertension and no pre-existing hypertension documented, using Chi-squared test

*ACEi* angiotensin converting enzyme inhibitor, *ARB* angiotensin receptor blocker, *BB* beta-blocker, *CCB* calcium channel blocker, *D* diuretic. ACEi or ARB is a pooled variable and distinct from ACEi and ARB

In-hospital mortality occurred in (*n* = 2020, 22.0%), with a greater number of deaths in patients with pre-existing hypertension (*n* = 1153) versus those without hypertension (*n* = 867). The number of palliative discharges and transfers to other facility was similar in those with and without hypertension (Table 4). The proportion of in-hospital mortality was similar between groups up to the age group 60–69 years, beyond this, the proportion of patients with pre-existing hypertension and in-hospital mortality was greater (Supplementary Table 7). The number of days in hospital was similar between groups with a greater proportion of patients without pre-existing hypertension admitted to ICU (22.7 vs. 18.6%; *p* < 0.001) compared to those with pre-existing hypertension. However, the number of days spent in ICU was similar (12 vs. 12 days; *p* > 0.9).

**Table 4:** Outcomes from hospital admission, stratified by pre-existing hypertension

		Pre-existing hypertension		
Characteristic	Overall, *N* = 9197	No, *N* = 4754 (51.7%)	Yes, *N* = 4443 (48.3%)	*p* value
In-hospital mortality	2020 (22.0%)	867 (18.2%)	1153 (26.0%)	<0.001
Discharged alive	6498 (70.7%)	3524 (74.1%)	2974 (66.9%)	<0.001
Discharged alive- Palliative	68 (0.7%)	37 (0.8%)	31 (0.7%)	0.7
Transfer to other facility	611 (6.6%)	326 (6.9%)	285 (6.4%)	0.4
Length of stay in hospital, days	8 (4, 16)	8 (4, 16)	9 (5, 17)	<0.001
Admission to ICU	1908 (20.7%)	1,081 (22.7%)	827 (18.6%)	<0.001
Length of stay ICU, days	12 (6, 22)	12 (6, 23)	12 (6, 22)	>0.9
Non-invasive ventilation	986 (10.7%)	559 (11.8%)	427 (9.6%)	<0.001
Invasive ventilation	1568 (17.0%)	887 (18.7%)	681 (15.3%)	<0.001

*p* value is for comparison between pre-existing hypertension and no pre-existing hypertension documented, using Wilcoxon rank sum test or Chi-squared test

*ICU* intensive care unit

### Unadjusted analyses

In unadjusted analysis, increasing age was exponentially associated with greater odds of in-hospital mortality. When <50 years was used as the reference (Supplementary Table 8) the age group (60–69 years) was associated with an increased (OR 4.23, 95% CI 3.23, 5.64) for in-hospital mortality. The univariate analyses for comorbidities using non-imputed data are shown in Supplementary Fig. 1.

### Primary outcome

#### Adjusted analyses

Although in unadjusted analysis, pre-existing hypertension was associated with greater odds for in-hospital mortality (OR 1.57, 95% CI 1.42,1.74), when adjusted for age and sex, this effect was substantially attenuated and became non-significant (aOR 1.06, 95% CI 0.95,1.18). Similar results were found in fully adjusted models (aOR 0.97, 95% CI 0.87,1.10).

### Secondary outcomes

#### Anti-hypertensive medication

**Fig. 2:**
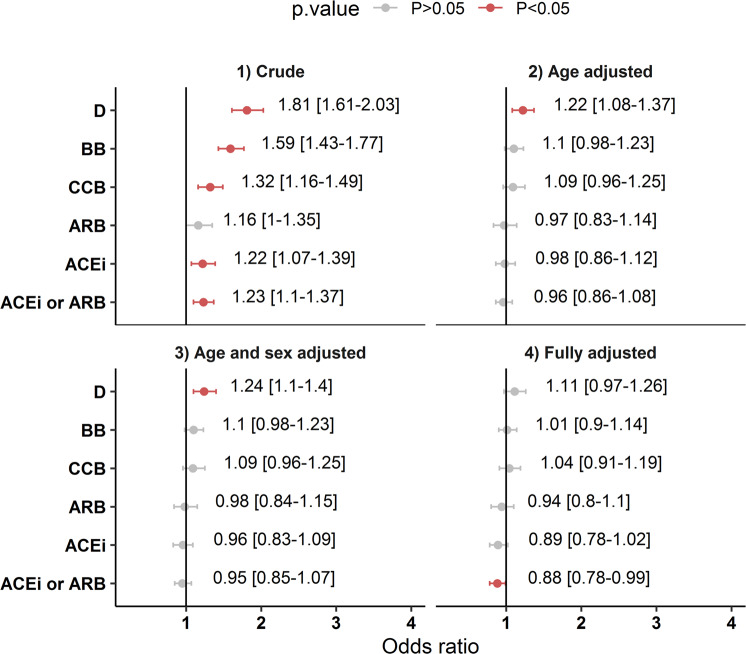
Association of anti-hypertensive medications and in-hospital mortality. Data displayed is odds ratio for (1) crude unadjusted or adjusted odds ratio [95% CI] *N* = 9197. Grey circle indicates *p* value > 0.05 and red circle indicates *p* value < 0.05. ACEi angiotensin converting enzyme inhibitor, ARB angiotensin receptor blocker, BB beta-blocker, CCB calcium channel blocker, D diuretic. ACEi or ARB is a pooled variable *N* = 2499 and was examined separately to ACEi *N* = 1512 and ARB *N* = 1007. Solid vertical line on x-axis 1 indicates reference—not receiving a type of anti-hypertensive medication

In unadjusted analysis, all classes of anti-hypertensive medications apart from ARBs (OR 1.16, 95% CI 1.00,1.35) were associated with an increased probability of in-hospital mortality (Fig. 2). In fully adjusted models, this trend was markedly attenuated, with no association between any type of antihypertensive medication and increased mortality. Use of ACEi or ARBs when pooled in these fully adjusted models tended towards having a protective effect (aOR 0.88, 95% CI 0.78,0.99).

#### Sensitivity analyses

In sensitivity analysis, those who were clinically suspected, but did not have a laboratory proven SARS-CoV-2 infection were excluded (*n* = 946, 10.3%). This resulted in similar findings to the primary analysis, where in fully adjusted models, pre-existing hypertension was not associated with increased mortality (aOR 0.98, 95% CI 0.87, 1.10). Similarly, when those prescribed a class of anti-hypertensive without a documented diagnosis of pre-existing hypertension, were excluded from the analysis (*n* = 985, 10.7%), this strengthened the association of pre-existing hypertension and increased outcomes in the unadjusted analysis (OR 1.90, 95% CI 1.70, 2.12). However, this effect was completely attenuated with full adjustment in line with findings from the primary analysis (aOR 0.99, 95% CI 0.87, 1.12).

## Discussion

Hypertension has been consistently reported as an important risk factor for more severe disease and mortality in Covid-19 patients admitted to the hospital, reiterated in the most recent scientific brief from the WHO [20]. Our study, confirms that pre-existing hypertension is the most common comorbidity in patients hospitalized with Covid-19. However, a definitive and important primary finding of this study was that whilst pre-existing hypertension was associated with in-hospital mortality in crude unadjusted analysis, when the analysis is fully adjusted for known confounders, especially age, this association is no longer apparent. This finding was robust in subsequent sensitivity analyses. Furthermore, unadjusted crude analysis of the impact of prior hypertension treatment demonstrated that all classes of anti-hypertensive medication, with the possible exception of ARBs, appeared to be associated with increased in-hospital mortality. However, this apparent adverse effect was completely attenuated in fully adjusted analyses and demonstrated that prior treatment with RAS blockers, i.e. ACEi or ARB showed a tendency towards a protective effect against mortality in hospitalised patients. Mindful of the commonality of hypertension, these findings have important implications for public health messaging with respect to the role of hypertension and its treatment in the context of Covid-19.

The current study comprised of individual patient level data from multiple centres across 12 countries, principally in the United Kingdom and Europe (Supplementary Table 10). In the study, 48.3% of patients had documented pre-existing hypertension and this prevalence was consistent with the age profile for the overall population studied. The proportion of people with pre-existing hypertension was greater than reported in previously published pooled data [9] (*n* = 48,317; 26.2%), which mainly included studies from China early in the pandemic. The proportion of patients with pre-existing hypertension in our study was however, less than that seen in another large, pooled study from the USA (*n* = 308,101, 66.9%) [14]. Differences between individual studies could be due to the definition used for pre-existing hypertension, the age of the cohort and the countries included for analysis.

### Pre-existing hypertension and outcomes

Previously published individual studies [1, 10–12, 14, 17, 18] and meta-analyses [9, 23] have demonstrated that without adjustment for any confounding, pre-existing hypertension is associated with an OR of 2–4 for mortality in patients hospitalized with Covid-19. In our study, we found pre-existing hypertension to be associated with a slightly lower risk for in-hospital mortality than in these previous studies, unadjusted (OR 1.57, 95% CI 1.42,1.74). Similar data to our findings were reported from one meta-analysis of older patients (>60 years) with pre-existing hypertension (OR 1.86, 95% CI 1.55, 2.23) [9]. However, in this analysis most study outcomes comprised a composite of severe Covid-19, admission to ICU or respiratory distress rather than in-hospital mortality. We considered whether the weaker unadjusted association of pre-existing hypertension with in-hospital mortality in our study, could be explained by inclusion of data beyond the first wave of the pandemic, when outcomes might have been improved by better treatment strategies. However, this is an unlikely explanation for the difference in adjusted mortality attributed to hypertension in different reports because >90% of hospital admissions in our database were in the first wave of the pandemic across the United Kingdom and Europe and within 3 months of 1st March 2020. Moreover, the OR for mortality associated with hypertension in unadjusted analyses, tended to rise and not decrease between patients admitted to hospital in the first and subsequent waves of the pandemic (Supplementary Table 9).

We restricted our mortality analysis to patients who died in the hospital they were admitted to, by excluding data from those who might have died subsequently, i.e. subsequent to palliative discharge and from people transferred to another facility. However, this represented only (*n* = 679, 7.4%) of patients and was equally distributed between those with and without pre-existing hypertension.

We found age to be a significant, exponential factor, for mortality in hospitalized Covid-19 patients, which is consistent with previous studies [15, 18, 24–26]. Furthermore, we and others [9] found a greater number of fatal outcomes in younger as opposed to older hypertensive patients in crude analysis. More severe, or a combination of additional comorbidities, beyond hypertension alone, may explain the different outcomes in older than younger individuals in our study. The tracking of major comorbidities with hypertension, as revealed by our data, particularly diabetes [2, 3] makes it difficult to elucidate independent risk factors and outcomes. However, in one large study [15], the risk of death was ~20-fold greater in those aged over 80 years compared to that in people aged 50–59 years with full adjustment. Despite other studies reporting a marked age effect in analysis [18, 25, 26], age was either incorporated in multivariable analysis [1, 18] and not analysed as a single factor, or not discussed [27, 28]. This has been a major factor leading to the erroneous conclusion that hypertension is an independent risk factor for mortality in patients with Covid-19

It is well recognised that in most populations, the prevalence of hypertension increases markedly with age, occurring in over 50% of patients after the age of 60 years. Thus, the powerful and exponential effect of age on mortality from Covid-19 was always a plausible explanation for the association between hypertension and mortality in these patients. Consistent with this hypothesis, when adjusting for age alone, we found that the odds ratio of 1.57 for mortality associated with pre-existing hypertension, was reduced to an (aOR 1.04, 95% CI 0.94,1.16), i.e., with this single adjustment alone, the association with hypertension was no longer significant. In smaller studies (*n* < 1000) that have used multivariable adjustment, pre-existing hypertension remained a significant risk factor for in-patient death; (aOR 1.61, 95% CI 1.10,2.61) [12], (aOR 1.88, 95% CI 1.01,3.55) [11] and poorer survival probability (aHR 2.12, 95% CI 1.17,3.82) [1], (aHR 1.80, 95% CI 1.20,2.70) [17]. In our study, in-hospital mortality occurred in 22.0% of the sample which represents a lower proportion than reported in studies by Basu (39.8%) and Khawaja (30.9%) who also had a greater proportion of people with hypertension, 69.5 and 53.4%, respectively. Gao and Pan reported lower proportions with pre-existing hypertension (~30%), than the present study together with younger populations with fewer outcomes. A combination of these factors may explain the variability in previous findings compared to the data reported in our study. Nevertheless, the larger scale of our study and the fact that it was specifically focused on collecting data on cardiovascular disease and risk factors, highlights the importance of our findings.

Larger individual studies (*n* > 1000) found no association of pre-existing hypertension with ICU mortality (aHR 0.99, 95% CI 0.81,1.22) [18], or in-hospital death (aOR 1.2, 95% CI 0.69,2.25) [27]. Similarly, in one very large study (~*n* = 400,000), no association between hypertension and mortality was seen, after multivariate adjustment (aRR 1.09, 95% CI 0.71,1.67) [29]. Taken together, we can conclude that smaller studies *n* < 1000, more often report that pre-existing hypertension, even after adjustment for confounders, is associated with worse outcomes from Covid-19, whereas studies with larger sample sizes tend to show no such association. It has been particularly challenging to evaluate this association with meta-analyses, as noted by the WHO scientific brief, because of a lack of patient level data and heterogenous definitions and methods for outcome ascertainment and the heterogeneity of patients included in the component analyses. Thus, a single large study such as ours, with patient level data, is likely to provide a more reliable estimate of the association between pre-existing hypertension and death from Covid-19, at least for hospitalized patients.

### Impact of anti-hypertensive medications

In our study, prior treatment with all major classes of anti-hypertensive medication was associated with increased risk of in-hospital mortality from Covid-19 in crude unadjusted analysis, with the possible exception of ARBs which was borderline (OR 1.16, 95% CI 1.00,1.35). However, with full adjustment, these apparent associations were lost and showed a tendency in pooled data for treatment with either ACEi or ARB, to have a protective effect against mortality (aOR 0.88, 95% CI 0.78,0.99). This finding is consistent with other reports indicating that prior treatment with an ACEi or ARB may show a trend towards some protective effect against in-hospital mortality in individual studies [27] ACEi or ARB (aOR 0.75, 95% CI 0.43,1.3) (aHR 0.79, 95% CI 0.51,1.15) [30] or no effect (aOR 0.99, 95% CI 0.83,1.18) [31]. Larger pooled analyses that examine the effect ACEi or ARB on in-hospital mortality show results in the same direction but with a greater magnitude of effect in adjusted outcomes 0.57–0.65 [32, 33]. A key finding of our study was that for the >25% of patients receiving ACEi or ARB prior to developing Covid-19, we found no definitive evidence of an adverse association with mortality or harm with prior ACEi or ARB treatment, and if anything, a trend towards benefit.

There were 985 (10.7%) patients without documented pre-existing hypertension who were receiving a class of medication that could be used to treat hypertension. The prevalence of cardiac and kidney disease in this sub-group was 6 and 5-fold greater than in those with no documented pre-existing hypertension and not receiving a class of anti-hypertensive medication, respectively (Supplementary Table 5). The most common classes of medication were BB, D and ACEi and importantly, the sub-class of these medications is in line with the prevalence of and treatment for heart failure, arrythmias and chronic kidney disease in this group (Supplementary Table 4). To ensure the risk profile of this group did not mask the effect of documented pre-existing hypertension nor anti-hypertensive medications on in-hospital mortality, sensitivity analysis demonstrated that their removal from the analysis did not alter the main findings (Supplementary Figs. 2 and 3).

### Symptoms at hospital admission- influence of hypertension

We also examined the association between pre-existing hypertension and Covid-19 symptoms at the time of hospital admission. The most common symptoms reported on admission to hospital were a fever, cough and dyspnoea. Those with pre-existing hypertension had a lower prevalence of these symptoms and this is most likely related to the older age of patients with pre-existing hypertension. In our study, those with pre-existing hypertension had a median age of 9 years older (73 vs. 62 years) and previous studies have suggested that older adults >70 years have lower prevalence of these classic Covid-19 symptoms than younger adults 30–60 years with Covid-19 [34]. Conversely, another study [1] reported that pre-existing hypertensive patients had greater occurrence of classic symptoms, despite a similar age profile to our study. It is difficult to reconcile these differences, however, that study was much smaller, had a younger hypertensive cohort and a much lower prevalence of pre-existing hypertension (29.6%) than our study.

Our study has several strengths. The study is large with a high number of in-hospital deaths, providing significant power for the analyses. Unlike many prior cohort studies of Covid-19, this study and database were designed by cardiovascular clinical scientists and specifically designed to evaluate cardiovascular risk factors, comorbidities and disease on outcomes from Covid-19. As such, there was a particular focus on ensuring relevant data was collected and curated at multiple centres and countries using a structured data collection instrument. This provided high quality patient-level data and conferred homogeneity of the dataset, which is not usually the case in meta-analysis or other pooled analyses. Where data was missing or imputed, or when other sensitivity analyses were conducted, this did not alter the key findings of this study.

Our study also has some limitations. Firstly, pre-existing hypertension could be underrepresented in those who have not had regular medical consultations and in whom hypertension is undiagnosed. This would depend on individual countries’ strategies for screening and management of hypertension. Secondly, we have no data on the duration of pre-existing hypertension or quality of blood pressure control. As such, we were not able to differentiate between newly diagnosed versus chronic hypertension, which may confer a different risk profile. Thirdly, although the general missingness of data was low, especially considering the context for data collection, i.e. during the clinical pressure of a pandemic, one important risk factor; BMI, had a notable amount of missing data, i.e. >30% and imputation was used to overcome this. However, the imputation error was estimated to be ± 5.8 kg/m^2^ and this may have dampened the true impact of obesity in fully adjusted models.

## Conclusions

Reporting from a large and comprehensive database of patients hospitalized with Covid-19 in multiple centres across the United Kingdom and Europe, our data demonstrates that after appropriate adjustment for confounding, pre-existing hypertension, or treatment for hypertension, does not independently confer an increased risk of death from Covid-19 in hospitalised patients. Moreover, contrary to initial concerns, our data suggests that prior treatment of hypertensive patients with ACEi or ARB medication, rather than increasing risk, may actually confer some protection against in-hospital mortality. These findings have important public health implications. The constant reference to, and misrepresentation of hypertension as a major risk factor, or cause of severe disease or death from Covid-19, has caused unnecessary alarm amongst patients. Our data clearly shows that the relationship between hypertension and risk from Covid-19 is entirely explained by the co-segregation of hypertension and/or its treatments, with the real risk factors for adverse outcomes from Covid-19, notably; increased age and comorbidities such as; obesity, diabetes, chronic kidney disease and heart failure in particular.

## Supplementary information

Supplementary Information file
