# Supplementary material for: The impact of pre-existing hypertension and its treatment on outcomes in patients admitted to hospital with COVID-19
Source: Hypertens Res. 2022 Mar 29;45(5):834–45. doi: 10.1038/s41440-022-00893-5 (PMC8963889; doi:10.1038/s41440-022-00893-5)
Supplement: Supplementary file 1 — Supplementary Information file [file 41440_2022_893_MOESM1_ESM.docx]

**Supplementary Data**

**The impact of pre-existing hypertension and its treatment on outcomes in patients admitted to hospital with COVID-19**

McFarlane E^1^, Linschoten M^2^, Asselbergs FW^1,2,3,4^, Lacy PS^1^, Jedrzejewski D^1^, and Williams B^1,2^ on Behalf of the CAPACITY-COVID Consortium.

| **Supplementary Table 1: Missingness of data and imputation errors from random forest algorithm** | | | |
| --- | --- | --- | --- |
| **Variable** | **Error** | **Error name** | **Missingness, %** |
| ***Body mass index, kg/m^2^** | 33.52 | RMSE | 33.6 |
| **Peripheral artery disease** | 6 | PFC | 23.2 |
| **Dyslipidemia** | 30 | PFC | 5.8 |
| ***Chronic obstructive pulmonary disease** | 14 | PFC | 1.0 |
| ***Chronic kidney disease** | 11 | PFC | 0.9 |
| ***Diabetes - any type** | 27 | PFC | 0.6 |
| **Cardiac disease** | 1 | PFC | 0.5 |
| **Arrhythmia** | 11 | PFC | 0.5 |
| ***Heart failure** | 7 | PFC | 0.5 |
| **Coronary artery disease** | 9 | PFC | 0.5 |
| **Valvular heart disease** | 5 | PFC | 0.5 |
| **Congenital heart disease** | 0 | PFC | 0.5 |
| **Other heart disease** | 3 | PFC | 0.5 |
| RMSE; root mean squared error, PFC; percentage false classification.* variables used in fully adjusted models. | | | |

| **Supplementary Table 2: Demographics using imputed data set, stratified by pre-existing hypertension** | | | | |
| --- | --- | --- | --- | --- |
|  |  | **Pre-existing hypertension** | |  |
| **Characteristic** | **Overall**, N = 9,197 | **No**, N = 4,754 (51.7%) | **Yes**, N = 4,443 (48.3%) | **p-value** |
| **Sex, Male** | 5,573 (60.6%) | 2,996 (63.0%) | 2,577 (58.0%) | <0.001 |
| **Age, Years** | 69 (57, 78) | 62 (51, 74) | 73 (63, 81) | <0.001 |
| **Age Group, Years** |  |  |  | <0.001 |
| < 50 | 1,298 (14.1%) | 1,088 (22.9%) | 210 (4.7%) |  |
| 50-59 | 1,531 (16.6%) | 989 (20.8%) | 542 (12.2%) |  |
| 60-69 | 1,937 (21.1%) | 980 (20.6%) | 957 (21.5%) |  |
| 70-79 | 2,321 (25.2%) | 924 (19.4%) | 1,397 (31.4%) |  |
| 80-89 | 1,736 (18.9%) | 626 (13.2%) | 1,110 (25.0%) |  |
| 90-100+ | 374 (4.1%) | 147 (3.1%) | 227 (5.1%) |  |
| **Body mass index (BMI), kg/m^2^** | 27.2 (24.2, 30.9) | 26.8 (23.9, 30.4) | 27.8 (24.6, 31.6) | <0.001 |
| **Obese, BMI ≥ 30 kg/m^2^** | 1,877 (30.7%) | 832 (26.9%) | 1,045 (34.7%) | <0.001 |
| **Heart disease – any type** | 3,208 (35.1%) | 1,028 (21.8%) | 2,180 (49.3%) | <0.001 |
| Arrhythmia or conduction disorder | 1,392 (15.2%) | 492 (10.4%) | 900 (20.3%) | <0.001 |
| Heart failure | 667 (7.3%) | 179 (3.8%) | 488 (11.0%) | <0.001 |
| Coronary artery disease | 1,356 (14.8%) | 408 (8.6%) | 948 (21.4%) | <0.001 |
| Valvular heart disease | 408 (4.5%) | 143 (3.0%) | 265 (6.0%) | <0.001 |
| Congenital heart disease | 35 (0.4%) | 17 (0.4%) | 18 (0.4%) | 0.6 |
| Other heart disease | 466 (5.1%) | 110 (2.3%) | 356 (8.0%) | <0.001 |
| **Chronic Kidney Disease** | 1,123 (12.3%) | 290 (6.2%) | 833 (18.9%) | <0.001 |
| **Peripheral artery disease** | 359 (5.1%) | 115 (3.1%) | 244 (7.4%) | <0.001 |
| **Chronic obstructive pulmonary disease** | 1,052 (11.6%) | 450 (9.6%) | 602 (13.7%) | <0.001 |
| **Diabetes - (Type 1 or 2)** | 2,391 (26.2%) | 764 (16.1%) | 1,627 (37.0%) | <0.001 |
| **Dyslipidemia** | 2,973 (34.3%) | 845 (18.7%) | 2,128 (51.5%) | <0.001 |
| **Ethnicity** |  |  |  | <0.001 |
| Arab | 494 (5.4%) | 290 (6.1%) | 204 (4.6%) |  |
| Asian | 623 (6.8%) | 358 (7.5%) | 265 (6.0%) |  |
| Black | 292 (3.2%) | 141 (3.0%) | 151 (3.4%) |  |
| Latin American | 23 (0.3%) | 11 (0.2%) | 12 (0.3%) |  |
| Other | 488 (5.3%) | 304 (6.4%) | 184 (4.1%) |  |
| Unknown | 964 (10.5%) | 541 (11.4%) | 423 (9.5%) |  |
| White | 6,313 (68.6%) | 3,109 (65.4%) | 3,204 (72.1%) |  |
| p-value is for comparison between pre-existing hypertension and no documented pre-existing hypertension, using Wilcoxon rank sum test or Chi-squared test. | | | | |

| **Supplementary Table 3: Symptoms reported at admission to hospital, stratified by pre-existing hypertension** | | | | |
| --- | --- | --- | --- | --- |
|  |  | **Pre-existing hypertension** | |  |
| **Characteristic** | **Overall**, N = 9,197 | **No**, N = 4,754 (51.7%) | **Yes**, N = 4,443 (48.3%) | **p-value** |
| **Fever >38.0°c** | 5,611 (61.0%) | 3,015 (63.4%) | 2,596 (58.4%) | <0.001 |
| **Dyspnea** | 5,276 (57.4%) | 2,790 (58.7%) | 2,486 (56.0%) | 0.008 |
| **Cough** | 5,206 (56.6%) | 2,819 (59.3%) | 2,387 (53.7%) | <0.001 |
| **Fatigue** | 2,922 (31.8%) | 1,522 (32.0%) | 1,400 (31.5%) | 0.6 |
| **Gastrointestinal** | 2,122 (23.1%) | 1,115 (23.5%) | 1,007 (22.7%) | 0.4 |
| **Chest pain** | 809 (8.8%) | 447 (9.4%) | 362 (8.1%) | 0.034 |
| **Sore throat** | 682 (7.4%) | 392 (8.2%) | 290 (6.5%) | 0.002 |
| **Loss of smell** | 341 (3.7%) | 215 (4.5%) | 126 (2.8%) | <0.001 |
| **Near (syncope)** | 330 (3.6%) | 149 (3.1%) | 181 (4.1%) | 0.015 |
| **Orthopnea** | 132 (1.4%) | 64 (1.3%) | 68 (1.5%) | 0.5 |
| **Palpitations** | 104 (1.1%) | 48 (1.0%) | 56 (1.3%) | 0.3 |
| **Peripheral edema** | 73 (0.8%) | 17 (0.4%) | 56 (1.3%) | <0.001 |
| p-value is for comparison between pre-existing hypertension and no pre-existing hypertension documented, using Chi-squared test. | | | | |

| **Supplementary Table 4: Anti-hypertensive medication - sub class, stratified by pre-existing hypertension** | | |
| --- | --- | --- |
|  | **Pre-existing Hypertension** | |
| **Characteristic** | **No**, N = 4,754 (51.7%) | **Yes**, N = 4,443 (48.3%) |
| **ACEi** | **286 (6.0%)** | **1226 (27.6%)** |
| Perindopril | 108 (2.3%) | 321 (7.2%) |
| Lisinopril | 54 (1.1%) | 298 (6.7%) |
| Ramipril | 64 (1.3%) | 271 (6.1%) |
| Enalapril | 35 (0.7%) | 262 (5.9%) |
| Fosinopril | 12 (0.3%) | 19 (0.4%) |
| Captopril | 7 (0.1%) | 22 (0.5%) |
| Unknown | 3 (0.1%) | 18 (0.4%) |
| Other | 3 (0.1%) | 15 (0.3%) |
| **ARB** | **118 (2.5%)** | **889 (20.0%)** |
| Losartan | 54 (1.1%) | 442 (9.9%) |
| Ibersartan | 24 (0.5%) | 159 (3.6%) |
| Candesartan | 20 (0.4%) | 131 (2.9%) |
| Valsartan | 15 (0.3%) | 102 (2.3%) |
| Telmisartan | 2 (0%) | 28 (0.6%) |
| Olmesartan | 1 (0%) | 21 (0.5%) |
| Other | 1 (0%) | 6 (0.1%) |
| Unknown | 1 (0%) | 0 (0%) |
| **CCB** | **174 (3.7%)** | **1,324 (29.8%)** |
| Amlodipine | 121 (2.5%) | 1,041 (23.4%) |
| Nifedipine | 16 (0.3%) | 119 (2.7%) |
| Lercanidipine | 9 (0.2%) | 57 (1.3%) |
| Barnidipine | 6 (0.1%) | 45 (1%) |
| Felodipine | 4 (0.1%) | 21 (0.5%) |
| Diltiazem | 10 (0.2%) | 17 (0.4%) |
| Other | 7 (0.1%) | 16 (0.4%) |
| Unknown | 1 (0%) | 8 (0.2%) |
| **D** | **381 (8%)** | **1,520 (34.2%)** |
| Bumetadine | 51 (1.1%) | 155 (3.5%) |
| Chlortalidone | 2 (0%) | 29 (0.7%) |
| Furosemide | 245 (5.2%) | 645 (14.5%) |
| Hydrochlorthiazide | 46 (1%) | 512 (11.5%) |
| Other | 37 (0.8%) | 179 (4%) |
| **BB** | **574 (12.1%)** | **1,828 (41.1%)** |
| Atenolol | 16 (0.3%) | 116 (2.6%) |
| Bisoprolol | 222 (4.7%) | 722 (16.3%) |
| Carvedilol | 16 (0.3%) | 47 (1.1%) |
| Labetalol | 0 (0%) | 7 (0.2%) |
| Metoprolol | 275 (5.8%) | 798 (18%) |
| Nebivolol | 12 (0.3%) | 78 (1.8%) |
| Propranolol | 19 (0.4%) | 26 (0.6%) |
| Other | 14 (0.3%) | 34 (0.8%) |
| ACEi; angiotensin converting enzyme inhibitor, ARB; angiotensin receptor blocker, BB; beta-blocker, CCB; calcium channel blocker, D; diuretic. | | |

| **Supplementary Table 5: Cardiovascular risk factors in those without pre-existing hypertension, stratified by class of anti-hypertensive medication** | | | |
| --- | --- | --- | --- |
|  | **Prescribed a class of anti-hypertensive medication** | |  |
| **Characteristic** | **No**, N = 3,769 | **Yes**, N = 985 | **p-value** |
| **Age, Years** | 59 (48, 71) | 74 (65, 82) | <0.001 |
| **Cardiac disease-any type** | 367 (9.7%) | 661 (67.1%) | <0.001 |
| Arrhythmia or conduction disorder | 174 (4.6%) | 318 (32.3%) | <0.001 |
| Heart failure | 32 (0.8%) | 147 (14.9%) | <0.001 |
| Coronary artery disease | 116 (3.1%) | 292 (29.6%) | <0.001 |
| Valvular heart disease | 53 (1.4%) | 90 (9.1%) | <0.001 |
| Congenital heart disease | 6 (0.2%) | 11 (1.1%) | <0.001 |
| Other heart disease | 50 (1.3%) | 60 (6.1%) | <0.001 |
| **Diabetes - (Type 1 or 2)** | 482 (12.8%) | 285 (28.9%) | <0.001 |
| **Chronic Kidney Disease** | 128 (3.4%) | 165 (16.8%) | <0.001 |
| **Chronic obstructive pulmonary disease** | 275 (7.3%) | 176 (17.9%) | <0.001 |
| **Peripheral artery disease** | 54 (1.4%) | 61 (6.2%) | <0.001 |
| **Dyslipidemia** | 501 (13.3%) | 385 (39.1%) | <0.001 |
| p-value is for comparison between pre-existing hypertension and no pre-existing hypertension documented, using Wilcoxon rank sum test or Chi-squared test. | | | |

| **Supplementary Table 6: Anti-hypertensive medication by age and pre-existing hypertension** | | | | | | |
| --- | --- | --- | --- | --- | --- | --- |
|  |  | **Age < 69 years** | | **Age > 69 years** | |  |
| **Characteristic** | **Overall**, N = 9,197 | **No HTN**,  N = 2,969 | **HTN**,  N = 1,607 | **No HTN**,  N = 1,785 | **HTN**,  N = 2,836 | **p-value** |
| **Prescribed anti-hypertensive** | 4,570 (49.7%) | 317 (10.7%) | 1,237 (77.0%) | 668 (37.4%) | 2,348 (82.8%) | <0.001 |
| **Number of anti-hypertensives** |  |  |  |  |  | <0.001 |
| 0 | 4,627 (50.3%) | 2,652 (89.3%) | 370 (23.0%) | 1,117 (62.6%) | 488 (17.2%) |  |
| 1 | 1,988 (21.6%) | 202 (6.8%) | 539 (33.5%) | 367 (20.6%) | 880 (31.0%) |  |
| 2 | 1,666 (18.1%) | 86 (2.9%) | 460 (28.6%) | 227 (12.7%) | 893 (31.5%) |  |
| 3 or more | 916 (10.0%) | 29 (1.0%) | 238 (14.8%) | 74 (4.1%) | 575 (20.3%) |  |
| **ACEi or ARB** | 2,499 (27.2%) | 143 (4.8%) | 792 (49.3%) | 260 (14.6%) | 1,304 (46.0%) | <0.001 |
| **Beta-blocker** | 2,391 (26.0%) | 176 (5.9%) | 523 (32.5%) | 395 (22.1%) | 1,297 (45.7%) | <0.001 |
| **Diuretic** | 1,806 (19.6%) | 80 (2.7%) | 388 (24.1%) | 283 (15.9%) | 1,055 (37.2%) | <0.001 |
| **CCB** | 1,498 (16.3%) | 66 (2.2%) | 503 (31.3%) | 108 (6.1%) | 821 (28.9%) | <0.001 |
| **ACEi** | 1,512 (16.4%) | 97 (3.3%) | 461 (28.7%) | 189 (10.6%) | 765 (27.0%) | <0.001 |
| **ARB** | 1,007 (10.9%) | 46 (1.5%) | 337 (21.0%) | 72 (4.0%) | 552 (19.5%) | <0.001 |
| *ACEi; angiotensin converting enzyme inhibitor, ARB; angiotensin receptor blocker, BB; beta-blocker, CCB; calcium channel blocker, D; diuretic.*, HTN; pre-existing hypertension.  p-value is for comparison between pre-existing hypertension and age group using Chi-squared test. | | | | | | |

| **Supplementary Table 7: In-hospital mortality stratified by age group and pre-existing hypertension** | | | | | |
| --- | --- | --- | --- | --- | --- |
|  | **No in-hospital mortality** | | **In-hospital mortality** | |  |
| **Characteristic** | **No HTN**, N = 3,887 | **HTN**,  N = 3,290 | **No HTN**,  N = 867 | **HTN**,  N = 1,153 | **p-value** |
| Age Group, Years |  |  |  |  | <0.001 |
| < 50 | 1,045 (27%) | 190 (5.8%) | 43 (5.0%) | 20 (1.7%) |  |
| 50-59 | 897 (23%) | 497 (15%) | 92 (11%) | 45 (3.9%) |  |
| 60-69 | 818 (21%) | 775 (24%) | 162 (19%) | 182 (16%) |  |
| 70-79 | 656 (17%) | 975 (30%) | 268 (31%) | 422 (37%) |  |
| 80-89 | 385 (9.9%) | 715 (22%) | 241 (28%) | 395 (34%) |  |
| 90-100+ | 86 (2.2%) | 138 (4.2%) | 61 (7.0%) | 89 (7.7%) |  |
| HTN; pre-existing hypertension.  p-value is for comparison of pre-existing hypertension and in-hospital mortality using Chi-squared test. | | | | | |

| **Supplementary Table 8: Crude association of age group and in-hospital mortality** | | | | |
| --- | --- | --- | --- | --- |
| **Characteristic** | **N** | **OR** | **95% CI** | **p-value** |
| Age Group, Years | 9,197 |  |  |  |
| < 50 | 1,298 | 1.00 |  |  |
| 50-59 | 1,531 | 1.93 | 1.42, 2.64 | <0.001 |
| 60-69 | 1,937 | 4.23 | 3.23, 5.64 | <0.001 |
| 70-79 | 2,321 | 8.29 | 6.39, 10.9 | <0.001 |
| 80-89 | 1,736 | 11.3 | 8.71, 15.0 | <0.001 |
| 90-100+ | 374 | 13.1 | 9.51, 18.3 | <0.001 |
| N: number, OR:odds ratio, CI: confidence interval. | | | | |

| **Supplementary Table 9: Crude association of pre-exisiting hypertension and in-hopsital mortality, stratified by date of hospital admission** | | | | | |
| --- | --- | --- | --- | --- | --- |
| Hospital admission date after | N | N events | OR | 95% CI | p-value |
| **1^st^ March 2020** | 9,197 | 2,020 | 1.57 | 1.42,1.74 | <0.001 |
| **1^st^ April 2020** | 5,434 | 1,138 | 1.56 | 1.37,1.78 | <0.001 |
| **1^st^ May 2020** | 1,695 | 305 | 1.91 | 1.49,2.47 | <0.001 |
| **1^st^ June 2020** | 732 | 118 | 2.93 | 1.95,4.45 | <0.001 |
| N: number, OR:odds ratio, CI: confidence interval. | | | | | |

| **Supplementary Table 10: Countries that contributed data for final analyses** |
| --- |
| Belgium |
| Egypt |
| France |
| Iran |
| Israel |
| Italy |
| Netherlands |
| Portugal |
| Russian Federation |
| Saudi Arabia |
| Spain |
| United Kingdom |


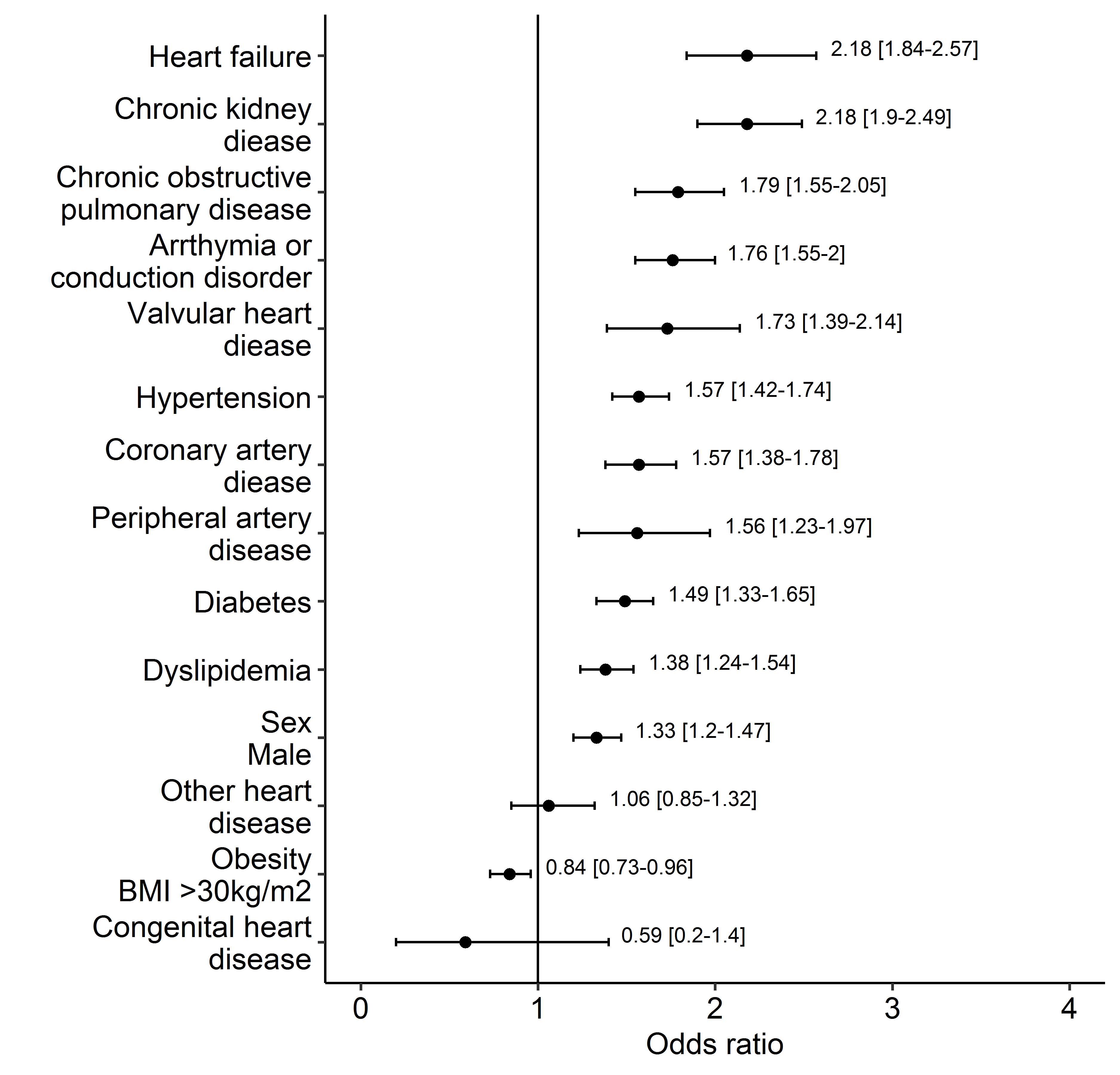


*Supplementary Figure 1: Univariate associations with in-hospital mortality using non-imputed data and logistic regression model. Text is odds ratio [95%CI]. Solid vertical line 1, indicates reference – no condition or comorbidity.*


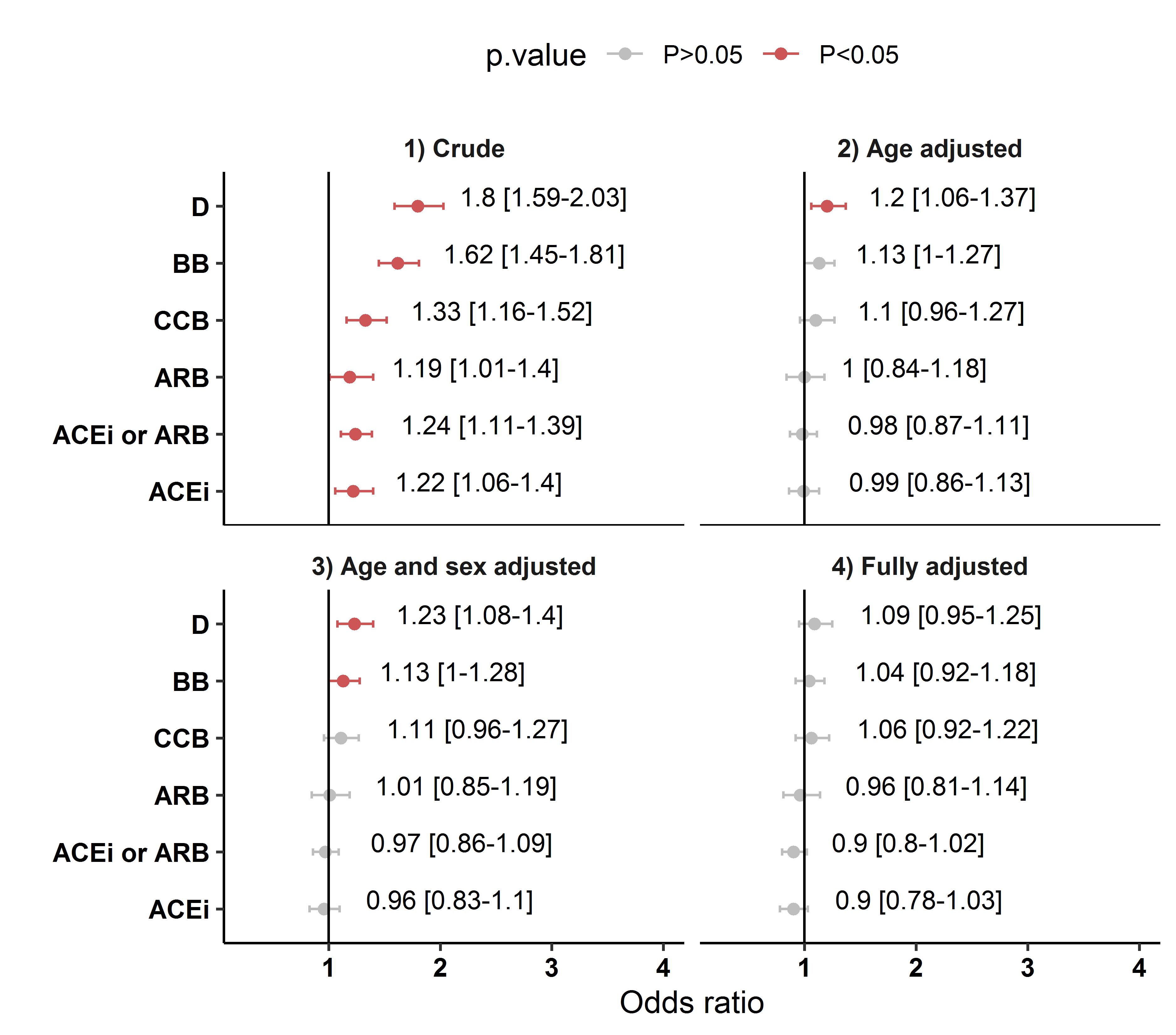


*Supplementary Figure 2: Association of anti-hypertensive medications and in-hospital mortality. Data used excludes (N = 946) those* *who did not have a positive Covid-19 test at or during hospital admission but who were highly suspected to have Covid-19 infection. Data displayed is odds ratio for 1) crude unadjusted or adjusted odds ratio [95%CI], N = 8,251. ACEi; angiotensin converting enzyme inhibitor, ARB; angiotensin receptor blocker, BB; beta-blocker, CCB; calcium channel blocker, D; diuretic. ACEi or ARB is a pooled variable N = 2,196 and was examined separately to ACEi N = 1,334 and ARB N= 881. Solid vertical line on x-axis 1 indicates reference – not receiving a type of anti-hypertensive medication.*


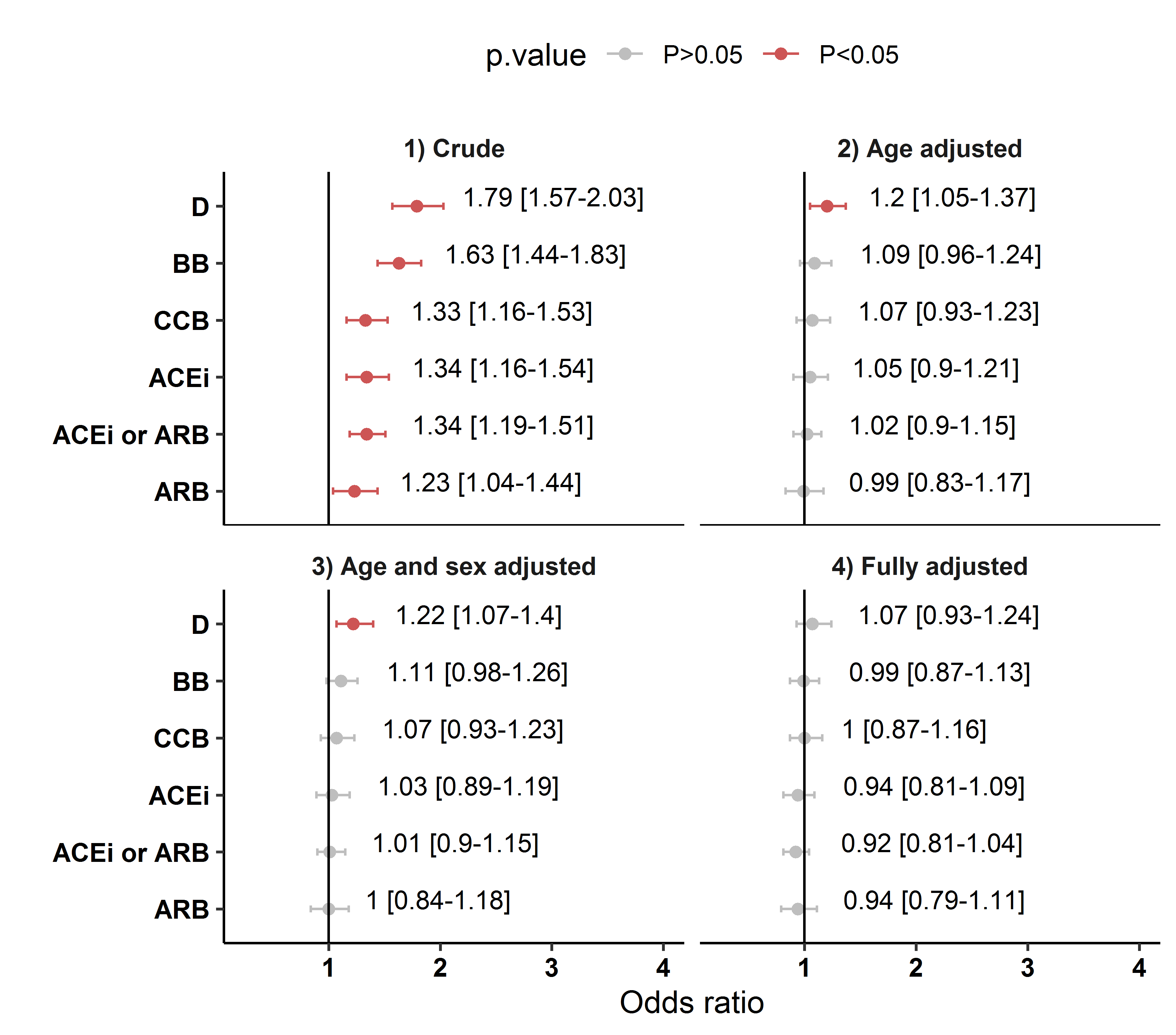


*Supplementary Figure 3: Association of anti-hypertensive medications and in-hospital mortality. Data used excludes (N = 985) those receiving a class of anti-hypertensive medication who did not have a diagnosis of pre-existing hypertension. Data displayed is odds ratio for 1) crude unadjusted or adjusted odds ratio [95%CI], N =8,212. ACEi; angiotensin converting enzyme inhibitor, ARB; angiotensin receptor blocker, BB; beta-blocker, CCB; calcium channel blocker, D; diuretic ACEi or ARB is a pooled variable N = 2,096 and was examined separately to ACEi N = 1,226 and ARB N=889. Solid vertical line on x-axis 1 indicates reference – not receiving a type of anti-hypertensive medication.*

**Collaborators within the CAPACITY-COVID consortium (listed alphabetically)**

Al-Ali AK^1^, Al-Muhanna FA^2^, Al-Windy NYY^3^, Almubarak YA^4^, Alnafie AN^5^, Alshahrani M^6^, Alshehri AM^7^, Anthonio RL^8^, Aujayeb A^12^, ten Berg JM^13^, van Boxem AJM^14^, Captur G^11,15^, Caputo M^16,17^, Charlotte N^18^, Dark P^19^, De Sutter J^20,21^, Delsing CE^22^, Dorman HGR^23^, Drost JT^24^, Emans ME^25^, Ferreira JB^26^, Gabriel L^27^, van Gilst WH^28^, Groenemeijer BE^29^, Haerkens-Arends HE^30^, van der Harst P^9^, Hedayat B^31^, van der Heijden DJ^32^, Hellou E^33^, Hermanides RS^34^, Hermans-van Ast JF^35^, van Hessen MWJ^36^, Heymans SRB^37,38,39^, van der Horst ICC^40,41^, van Ierssel SH^42^, Jewbali LS^43,44^, Kearney MT^45^, van Kesteren HAM^46^, Kietselaer BLJH^47^, Koning AMH^48^, Kopylov PY^49^, Kuijper AFM^50^, Kwakkel-van Erp JM^51^, van der Linden MMJM^52^, Linssen GCM^53^, Macias Ruiz R^54^, Magdelijns FJH^55^, Martens FMAC^56^, McCann GP^57^, van der Meer P^58^, Meijs MFL^59^, Messiaen P^60,61^, Monraats PS^62^, Montagna L^63^, Moriarty A^64^, Mosterd A^65^, Nierop PR^66^, van Ofwegen-Hanekamp CEE^67^, Pinto YM^68^, Poorhosseini H^69^, Prasad S^70,71^, Redón J^72,73^, Reidinga AC^74^, Ribeiro MIA^75^, Ripley DP^76^, Salah R^77^, Saneei E^78^, Saxena M^79^, Schaap J^80,81^, Schellings DAAM^82^, Schut A^80^, Shafiee A^83^, Shore AC^84^, Siebelink HJ^85^, van Smeden M^86^, Smits PC^87^, Pisters R^88^, Tessitore E^89^, Tieleman RG^28,90^, Timmermans P Jr^91^, Tio RA^92,93^, Tjong FVY^68,94,95^, den Uil CA^43,44,96^, Van Craenenbroeck EM^97^, van Veen HPAA^98^, Veneman T^99^, Verschure DO^100^, de Vries JK^101^, van de Wal RMA^102^, van de Watering DJ^103^, Westendorp ICD^104^, Westendorp PHM^105^, Weytjens C^106^, Wierda E^95^, Woudstra P^108^, Wu KW^109^, Zaal R^110^, Zaman AG^111^, van der Zee PM^112^

**Affiliations of Collaborators**

1. Department of Clinical Biochemistry, King Fahd Hospital of the University, Imam Abdulrahman Bin Faisal University, Alkhobar, Saudi Arabia
2. Department of Internal Medicine, King Fahd Hospital of the University, Imam Abdulrahman Bin Faisal University, Alkhobar, Saudi Arabia
3. Department of Cardiology, Gelre Hospital Zutphen, Zutphen, the Netherlands
4. Department of Critical Care, King Fahd Hospital of the University, Imam Abdulrahman Bin Faisal University, Alkhobar, Saudi Arabia
5. Department of Pathology, King Fahd Hospital of the University, Imam Abdulrahman Bin Faisal University, Alkhobar, Saudi Arabia
6. Department of Emergency Medicine, King Fahd Hospital of the University, Imam Abdulrahman Bin Faisal University, Alkhobar, Saudi Arabia
7. Department of Internal Medicine, Cardiology Section, King Fahd Hospital of the University, Imam Abdulrahman Bin Faisal University, Alkhobar, Saudi Arabia
8. Department of Cardiology, Treant Zorggroep, Emmen, the Netherlands
9. Department of Cardiology, Division of Heart and Lungs, University Medical Center Utrecht, Utrecht University, Utrecht, the Netherlands
10. Health Data Research United Kingdom and Institute of Health Informatics, University College London, London, United Kingdom
11. Institute of Cardiovascular Science, Faculty of Population Health Sciences, University College London, London, United Kingdom
12. Department of Respiratory and Acute Medicine, Northumbria Healthcare NHS Foundation Trust, Newcastle, United Kingdom
13. Department of Cardiology, St. Antonius Hospital, Nieuwegein, the Netherlands
14. Department of Pulmonology, Bravis Hospital, Roosendaal, the Netherlands
15. Department of Cardiology, Royal Free London NHS Foundation Trust, London, United Kingdo
16. Bristol Heart Institute, University Hospitals Bristol and Weston NHS Foundation Trust, Bristol, United Kingdom
17. Bristol Medical School, University of Bristol, Bristol, United Kingdom
18. Department of Cardiology, SSR Val Rosay, Saint Didier au Mont d'Or, Franc
19. Department of Critical Care, Salford Royal NHS Foundation Trust, Salford, United Kingdom
20. Department of Cardiology, AZ Maria Middelares, Ghent, Belgium
21. Department of Internal Medicine, Ghent University, Ghent, Belgium
22. Department of Internal Medicine and Infectious Diseases, Medisch Spectrum Twente, Enschede, the Netherlands
23. Department of Cardiology, Bravis Hospital, Roosendaal, the Netherlands
24. Department of Cardiology, Saxenburgh Medical Center, Hardenberg, the Netherlands
25. Department of Cardiology, Ikazia Hospital, Rotterdam, the Netherlands
26. Department of Cardiology, Hospital Professor Doutor Fernando Fonseca, Amadora, Portugal
27. Department of Cardiology, CHU UCL Namur site Godinne, Université Catholique de Louvain, Yvoir, Belgium
28. Department of Cardiology, University Medical Center Groningen, Groningen, the Netherlands
29. Department of Cardiology, Gelre Hospital Apeldoorn, Apeldoorn, the Netherlands
30. Department of Cardiology, Jeroen Bosch Hospital, 's-Hertogenbosch, the Netherlands
31. Department of Cardiology, Tehran Heart Center, Cardiovascular Diseases Research Institute, Tehran University of Medical Sciences, Tehran, Iran
32. Department of Cardiology, Haaglanden Medical Center, the Hague, the Netherlands
33. Department of Cardiology, E.M.M.S Hospital, Nazareth, Israel
34. Department of Cardiology, Isala Hospital, Zwolle, the Netherlands
35. Durrer Center, Netherlands Heart Institute, Utrecht, the Netherlands
36. Department of Cardiology, Groene Hart Hospital, Gouda, the Netherlands
37. Department of Cardiology, Cardiovascular Research Institute Maastricht (CARIM), Maastricht University Medical Center+, Maastricht, the Netherlands
38. Department of Cardiovascular Sciences, Center for Molecular and Vascular Biology, KU Leuven, Belgium
39. The Netherlands Heart Institute, Utrecht, the Netherlands
40. Department of Intensive Care, Maastricht University Medical Center+, Maastricht University, Maastricht, the Netherlands
41. Cardiovascular Research Institute Maastricht (CARIM), Maastricht University Medical Center, Maastricht, the Netherlands
42. Department of General Internal Medicine, Infectious Diseases and Tropical Medicine, Antwerp University Hospital, Antwerp, Belgium
43. Department of Cardiology, Erasmus MC University Medical Center, Rotterdam, the Netherlands
44. Department of Intensive Care, Erasmus MC University Medical Center, Rotterdam, the Netherlands
45. Leeds Institute for Cardiovascular and Metabolic Medicine, University of Leeds, Leeds, United Kingdom
46. Department of Cardiology, Admiraal de Ruyter Hospital, Goes, the Netherlands
47. Department of Cardiology, Zuyderland Medical Center, Heerlen, the Netherlands
48. Department of Gynaecology, Amstelland Hospital, Amstelveen, the Netherlands
49. World-Class Research Center Digital Biodesign and Personalized Healthcare, I.M. Sechenov First Moscow State Medical University, Sechenov University, Moscow, Russia
50. Department of Cardiology, Spaarne Gasthuis, Haarlem, the Netherlands
51. Department of Pulmonology, Antwerp University Hospital, University of Antwerp, Edegem, Belgium
52. Department of Cardiology, Franciscus Vlietland, Schiedam, the Netherlands
53. Department of Cardiology, Ziekenhuis Groep Twente (ZGT), Almelo, the Netherlands
54. Arrhythmias Unit, Department of Cardiology, Hospital Universitario Virgen de las Nieves, Granada, Spain
55. Department of Internal Medicine, Division of General Internal Medicine, Section Geriatric Medicine, Cardiovascular Research Institute Maastricht (CARIM), Maastricht University Medical Center+, Maastricht, the Netherlands
56. Department of Cardiology, Deventer Hospital, Deventer, the Netherlands
57. Department of Cardiovascular Sciences, University of Leicester and Cardiovascular Theme, National Institute for Health Research (NIHR) Leicester Biomedical Research Center, Glenfield Hospital, Leicester, United Kingdom
58. Department of Cardiology, LangeLand Hospital, Zoetermeer, the Netherlands
59. Department of Cardiology, Thorax Center Twente, Medisch Spectrum Twente, Enschede, the Netherlands
60. Department of Infectious Diseases & Immunity, Jessa Hospital, Hasselt, Belgium
61. Faculty of Medicine and Life Sciences, Hasselt University, Hasselt, Belgium
62. Department of Cardiology, Elizabeth-TweeSteden Hospital, Tilburg, the Netherlands
63. Department of Cardiology, A.O.U. San Luigi Gonzaga, Orbassano, Turin, Italy
64. Cardiovascular Research Unit, Craigavon Area Hospital, Southern Health and Social Care Trust, Portadown, Nothern Ireland
65. Department of Cardiology, Meander Medical Center, Amersfoort, the Netherlands
66. Department of Cardiology, Franciscus Gasthuis, Rotterdam, the Netherlands
67. Department of Cardiology, Diakonessenhuis, Utrecht, the Netherlands
68. Amsterdam University Medical Center, University of Amsterdam, Heart Center; Department of Clinical and Experimental Cardiology, Amsterdam Cardiovascular Sciences, Amsterdam, the Netherlands
69. Department of Interventional Cardiology, Tehran Heart Center, Cardiovascular Diseases Research Institute, Tehran University of Medical Sciences, Tehran, Iran
70. National Heart and Lung Institute, Imperial College, London, United Kingdom
71. Royal Brompton Hospital, London, United Kingdom
72. Department of Internal Medicine, Clinic University Hospital, INCLIVA Health Research Institute, Valencia, Spain
73. Department of Medicine, School of Medicine, University of Valencia, Valencia, Spain
74. Department of Intensive Care, Martini Hospital, Groningen, the Netherlands
75. Intensive Care Unit, Hospital do Espírito Santo, Évora, Portugal
76. Department of Cardiology, Northumbria Healthcare NHS Foundation Trust, Newcastle, United Kingdom
77. Benha Faculty of Medicine, Benha, Egypt
78. Department of Nursing, Tehran Heart Center, Cardiovascular Diseases Research Institute, Tehran University of Medical Sciences, Tehran, Iran
79. Barts National Institute for Health Research (NIHR) Biomedical Research Center, William Harvey Research Institute, Queen Mary University of London, United Kingdom
80. The Dutch Network for Cardiovascular Research (WCN), Utrecht, the Netherlands
81. Department of Cardiology, Amphia Hospital, the Netherlands
82. Department of Cardiology, Slingeland Hospital Doetinchem, the Netherlands
83. Department of Cardiovascular Research, Tehran Heart Center, Cardiovascular Diseases Research Institute, Tehran University of Medical Sciences, Tehran, Iran
84. National Institute for Health Research (NIHR) Exeter Clinical Research Facility, Royal Devon and Exeter Hospital and University of Exeter College of Medicine & Health, Exeter, United Kingdom
85. Department of Cardiology, Leiden University Medical Center, Leiden, the Netherlands
86. Julius Center for Health Sciences and Primary Care, University Medical Center Utrecht, Utrecht University, Utrecht, the Netherlands
87. Department of Cardiology, Maasstad Hospital, Rotterdam, the Netherlands
88. Department of Cardiology, Rijnstate Hospital, Arnhem, the Netherlands
89. Department of Cardiology, University Hospitals of Geneva, Geneva, Switzerland
90. Department of Cardiology, Martini Hospital, Groningen, the Netherlands
91. Department of Cardiology, Heart Center Hasselt, Jessa Hospital, Hasselt, Belgium
92. Department of Cardiology, Catharina Hospital, Eindhoven, the Netherlands
93. Department of Educational Development and Research in the Faculty of Health, Medicine and Life Sciences, Catharina Hospital, Eindhoven, the Netherlands
94. Department of Cardiology, Vrije Universiteit Amsterdam, Amsterdam Cardiovascular Sciences, Amsterdam, the Netherlands
95. Department of Cardiology, Dijklander Hospital, Hoorn, the Netherlands
96. Department of Intensive Care Medicine, Maasstad Hospital, Rotterdam, the Netherlands
97. Cardiovascular Research, Antwerp University and Cardiology, Antwerp University Hospital, Antwerp, Belgium
98. Department of Pulmonology, Medisch Spectrum Twente, Enschede, the Netherlands
99. Department of Intensive Care, Ziekenhuis Groep Twente (ZGT), Almelo, the Netherlands
100. Department of Cardiology, Zaans Medical Center, Zaandam, the Netherlands
101. Department of Internal Medicine, Antonius Hospital, Sneek, the Netherlands
102. Department of Cardiology, Bernhoven Hospital, Uden, the Netherlands
103. Department of Cardiology, Albert Schweitzer Hospital, Dordrecht, the Netherlands
104. Department of Cardiology, Rode Kruis Hospital, Beverwijk, the Netherlands
105. Department of Cardiology, Beatrix Hospital, Gorinchem, the Netherlands
106. Department of Cardiology, CHVZ, University Hospital Brussels, Jette, Belgium
107. National Institute for Health Research Biomedical Research Center, University College London Hospitals, London, United Kingdom
108. Department of Cardiology, Medical Center Leeuwarden (MCL), Leeuwarden, the Netherlands
109. Department of Cardiology, van Weel-Bethesda Hospital, Dirksland, the Netherlands
110. Department of Pulmonology, Ziekenhuis Groep Twente (ZGT), Almelo, the Netherlands
111. Freeman Hospital, Newcastle Upon Tyne NHS Hospitals Foundation Trust and Newcastle University, Newcastle Upon Tyne, NE7 7DN, United Kingdom
112. Department of Cardiology, St. Jansdal Hospital, Harderwijk, the Netherlands
